# Vaping habits and respiratory symptoms using a smartphone app platform

**DOI:** 10.1186/s12889-024-19439-0

**Published:** 2024-07-30

**Authors:** Mi-Sun S. Lee, Ki-Do Eum, Joseph G. Allen, Jukka-Pekka Onnela, David C. Christiani

**Affiliations:** 1 Environmental and Occupational Medicine and Epidemiology Program, Department of Environmental Health, Harvard T.H. Chan School of Public Health, 665 Huntington Ave, Building I Room 1406A, Boston, MA 02115 USA; 2 Ariadne Labs at Brigham and Women’s Hospital, Harvard T.H. Chan School of Public Health, Boston, MA USA; 3 Department of Civil and Environmental Engineering, School of Engineering, Tufts University, Medford, MA USA; 4 Exposure, Epidemiology, and Risk Program, Department of Environmental Health, Harvard T.H. Chan School of Public Health, Boston, MA USA; 5 Department of Biostatistics, Harvard T.H. Chan School of Public Health, Boston, MA USA; 6 Pulmonary and Critical Care Division, Department of Medicine, Massachusetts General Hospital, Harvard Medical School, Boston, MA USA

**Keywords:** E-cigarettes, Vaping, Repeated measures, Epidemiology, Respiratory health, App platforms, Electronic nicotine delivery devices

## Abstract

**Background:**

Widespread use of e-cigarette (EC) or vaping products causes respiratory disorders including the nationwide outbreak of e-cigarette or vaping product use-associated lung injury (EVALI) in 2019. Chronic adverse health effects are now being reported as well. To address this important public health issue, an innovative approach of epidemic control and epidemiologic study is required. We aimed to assess the association between short-term and long-term use of EC products and respiratory health in adults using smartphone app data.

**Methods:**

A population-based, repeated measures, longitudinal smartphone app study that performed 8-day survey participation over 60 days for each participant from August 2020 to March 2021, including 306 participants aged 21 years and older in the US. The participants were asked to complete the respiratory health questionnaire daily, weekly, and monthly on their smartphone app. We analyzed the association between vaping habits and respiratory health using generalized linear mixed models (GLMMs).

**Results:**

EC use in the previous 7 days was associated with frequent cough (OR: 5.15, 95% CI: 2.18, 12.21), chronic cough (OR: 3.92, 95% CI: 1.62, 9.45), frequent phlegm (OR: 3.99, 95% CI: 1.44, 11.10), chronic phlegm (OR: 3.55, 95% CI: 1.41, 8.96), episodes of cough and phlegm (OR: 4.68, 95% CI: 1.94, 11.28), mMRC grade 3–4 dyspnea (OR: 3.32, 95% CI: 1.35 to 8.13), chest cold (OR: 3.07, 95% CI: 1.29, 7.33), eye irritation (OR: 2.94, 95% CI: 1.34, 6.47) and nose irritation (OR : 2.02, 95% CI: 0.95, 4.30). Relatively long-term effects of the past 90 days EC use was associated with an increased risk of wheeze (OR: 3.04, 95% CI: 1.31, 7.03), wheeze attack (OR: 2.78, 95% CI: 1.07, 7.24), mMRC grade 3–4 dyspnea (OR: 2.54, 9% CI: 1.05 to 6.18), eye irritation (OR: 3.16, 95% CI: 1.49, 6.68), and eye irritation during the past month (OR: 3.50, 95% CI: 1.52, 8.04).

**Conclusions:**

In this smartphone app-based repeated measures study, short-term and relatively long-term use of EC increased the risk of respiratory symptoms.

**Supplementary Information:**

The online version contains supplementary material available at 10.1186/s12889-024-19439-0.

## Background

The rapid implementation of mobile technology has been employed widely for health care and public health: chronic disease management, disease surveillance, and advances in the understanding of common diseases, particularly during the coronavirus disease 2019 (COVID-19) pandemic [1–4]. The smartphone applications (apps)-based approach is more feasible to contact and to recruit participants into research than are traditional approaches. It also allows researchers to collect and access the information in a timely manner. Epidemiologic studies have begun to take advantage of this immediacy to recruit study participants efficiently and to collect their information on potential hazardous exposures while simultaneously measuring health outcomes [5].

Widespread use of e-cigarette (EC) or vaping products has been shown as a potentially toxic inhalation that caused a national outbreak of severe lung illness and deaths, now was termed “EVALI” (e-cigarette, or vaping, product use-associated lung injury), in 2019 in the US (https://www.cdc.gov/tobacco/basic_information/e-cigarettes/severe-lung-disease.html). Previous studies found hazardous chemicals and microbial agents in e-cigarette (EC) or vaping products [6–10]. Chronic adverse health effects are now being reported as well [11–14]. However, limited epidemiological studies are available on assessing short-term and long-term effects of vaping habits on respiratory health.

In this repeated measures, longitudinal smartphone application (app)-based study, we aimed to assess the association between short-term and long-term use of EC products and respiratory health in adults in the United States. The results of the study may help develop effective vaping product-control policies concerning public safety and health.

## Methods

### Development of smartphone application (app) and participant onboarding

We developed a custom smartphone app, the Vaping and Health Study (VHS) app, as a new study platform to collect data on EC or vaping habits and respiratory health in the US population, working with app developers [Boston Technology Corporation (BTC), Marlborough, MA, USA] and our team (Supplementary Fig. 1). Prospective participants could download the VHS app from the Apple Store or the Google Play [15, 16]. The eligibility criteria for the study participation include: (1) individuals aged 21 years and older, legal age to buy tobacco products, including e-cigarettes based on Federal Tobacco 21 (or “T21”) law (https://www.fda.gov/tobacco-products/retail-sales-tobacco-products/tobacco-21), (2) literate in English, (3) all ethnic backgrounds, and (4) using a smartphone. We recruited individuals via posting to social networking service, such as the VHS Facebook page (https://www.facebook.com/VapingandHealthStudy/), the Harvard T.H. Chan School of Public Health (HSPH) Twitter, and also via the screening survey on our IRB-approved VHS Google Ads and Facebook Ads which are feasible and facilitate to recruit participants (Supplementary Fig. 2). [17] The screening survey includes information about the screening survey purpose (why the screening data are being collected, how the screening data will be used, and when the screening data will be deleted), eligibility questions, consent for the screening survey, and our contact email information. If individuals were determined eligible, they were informed that they are eligible for the study and emailed instructions to download the VHS app, and then proceed through the rest of the electronic consent (e-consent) process on their phone app. Once they signed the consent form, they were provided a PDF copy of their signed consent form. All protocols were reviewed and approved by the human research committees at the Harvard T.H. Chan School of Public Health (HSPH) (Protocol no. IRB20-0208**)**. All participants provided informed e-consent prior to participation.

### Study design

**Fig. 1:**
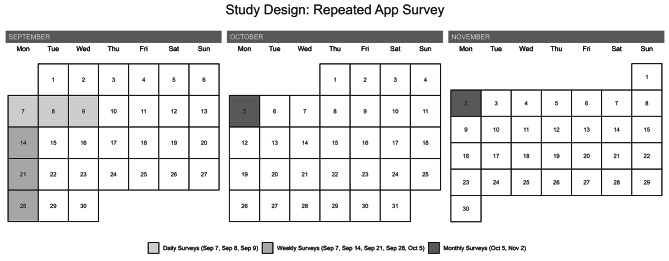
VHS Study Design (Example)

Our study is a repeated, longitudinal app-based study that performed 8 surveys participation over 60 days for each participant. The participants were asked to complete the survey daily, weekly, and monthly on their smartphone app. Daily surveys occurred three consecutive days in the first week of the survey; weekly surveys include a week, two weeks, and three weeks from the first day of survey, respectively; monthly surveys occurred on four weeks and eight weeks from the first day of survey, respectively. For example, if an enrolled subject starts to participate in the app survey, daily surveys are scheduled for September 7th (Survey 1: baseline), 8th (Survey 2), and 9th (Survey 3), weekly surveys are scheduled for September 7th (Survey 1), September 14 (Survey 4), September 21 (Survey 5), and September 28 (Survey 6), and monthly surveys are scheduled for September 7th (Survey 1), October 4th (Survey 7), and November 2nd (Survey 8) (Fig. 1). Over the course of the study, participants were sent a notification via the app every time. The participants were also informed that they can withdraw from the study anytime during the study period. Of the total 306 eligible individuals consented to participate in the study and provided a verified email address for enrollment, four participants later withdrew from the study, 40 participants did not submit the survey on their phone app, and 37 were missing in the baseline survey. Of the remaining 225 participants (73%) completed baseline survey (Survey 1), 220 with no missing covariate [missing age (*n* = 3) and other gender (*n* = 2)] were included in the final analysis.

### Respiratory symptoms

Information on respiratory symptoms using a modified version of the American Thoracic Society (ATS) division of lung disease questionnaire (ATS-DLD 1978) that contains frequent and chronic respiratory symptoms including cough, phlegm, wheeze, shortness of breath, and eyes and nose irritation was collected via the digital app data. The definitions and questions are given in Supplementary Table 1. Chronic cough is defined as cough that lasts > 3 months [18–20]. Chronic phlegm is defined as the production of phlegm that lasts > 3 months [21]. Shortness of breath was assessed based on the modified Medical Research Council (mMRC) dyspnea scale (grade 0–4) that it was scored by using the most severe response [22, 23].

### Assessment of EC habits

We measured short-term and long-term use of EC as a measure of EC habits. The ever use of EC was measured by asking, “Have you ever used an e-cigarette even one time?”, and respondents who reported ‘No’ were considered as never users [11, 24]. If the participant answered ‘Yes’, the information on short-term use of EC was measured by asking questions (1) “Have you ever used an e-cigarette within the past one day (within 24 hours)?”, (2) “Have you ever used an e-cigarette in the past seven days?”, and (3) “Have you ever used an e-cigarette in the past 30 days?”. The information on relatively long-term use of EC was measured by asking, “Have you ever used an e-cigarette in the past 90 days?”.

### Covariates

Based on previous literature and plausible associations between use of EC and respiratory outcomes, [25] covariates were considered a priori including age (years), sex (male and female), race/ethnicity (non-Hispanic White and others), education (college or above and high school), and smoking status (current, former, and never smoker). Covariates were assessed at baseline (Survey 1).

### Statistical analysis

Descriptive statistics were examined for all variables. Pearson Chi-square (χ^2^) test and two-tailed Fisher’s exact test were used to assess the differences in respiratory symptoms between EC ever user and never user groups. To assess the association between use of EC and respiratory symptoms, we analyzed the data using generalized linear mixed models (GLMMs) with random intercept (SAS^®^ PROC GLIMMIX; version 9.4, SAS Institute, Inc.). For the outcome of shortness of breath, five-level categorical shortness of breath (Grade 0–4) classified into two groups using the mMRC grade > 2 as the cutoff (mMRC grade 0–2 vs. mMRC grade 3–4). We ran the model to evaluate short-term and long-term effects of EC use on respiratory symptoms, adjusting for age (years), sex (male vs. female), race (non-Hispanic White vs. others), education (college or above vs. high school), and smoking status (current, former, and never smoker).

To assess the short-term effects of EC use on outcomes, up to three consecutive daily measurements were included in the analysis for daily effect and up to five consecutive weekly measurements with an interval of a week over four weeks were included in the analysis for weekly effect, respectively. To assess monthly and long-term effects, data of up to three consecutive monthly survey measurements and two consecutive bi-monthly survey measurements were included in the analysis for monthly and long-term effect, respectively. For example, daily effect using the surveys on Sep. 7 to 9; weekly effect using the surveys on Sep. 7, 14, 21, 28 and Oct. 5; monthly effect using the surveys on Oct 5. and Nov. 2; long-term effect was analyzed longitudinally the association between the past 90 days of EC use and respiratory symptoms using the surveys on Sep 7. and Nov. 2 **(**Fig. 1**)**. All statistical analyses were performed using SAS (version 9.4; SAS Institute, Inc.), R (version 4.2.2; R Development Core Team), and RStudio (version 2022.12.0 + 353) software.

## Results

The general baseline characteristics of the study population are presented in Table 1. The mean (SD) age was 33.8 (8.9); 133 (60.5%) were non-Hispanic White; 89 (40.0%) were males; 65 (29.6%) were current smokers; 120 (54.6%) were EC ever users; 56 (29.1%) of 120 were current EC users; and 100 (45.5%) were never EC users, respectively.

**Table 1:** Baseline characteristics of study participants (*N* = 220)

Variables	No. (%) or Mean ± SD
Age, years	33.8 ± 8.9
Race	
Non-Hispanic White	133 (60.5)
Others (Black, Hispanic, Asian, others)	87 (39.5)
Sex	
Male	89 (40.0)
Female	131 (60.0)
Education	
College or above	52 (23.6)
High school	168 (76.4)
Smoking status	
Current	65 (29.6)
Past	59 (26.8)
Never	96 (43.6)
EC use status	
Ever user	120 (54.6)
Nonuser	100 (45.4)

The baseline prevalence of respiratory outcomes by EC ever- and never users is shown in Table 2. Among the 220 participants included in the baseline analyses, the prevalence of frequent and chronic cough was 14.1% and 12.7%, respectively. The prevalence of frequent and chronic phlegm production was 15.5% and 16.8%, respectively; 12.3% report having episodes of cough and phlegm lasting > 3 weeks each year; 21.4% and 15.5% report wheeze and wheezing attack, respectively; 30 (13.6%) subjects had severe-to-very severe dyspnea (mMRC grade 3–4); 33.2% and 43.6% report chest cold and chest illnesses during the past 3 years, respectively; 25.9% and 19.6% report irritating eye and nose during the past months, respectively. Statistical differences between EC groups were found in frequent cough, frequent phlegm, wheeze, shortness of breath, chest cold symptoms.

**Table 2:** Baseline prevalence of respiratory symptoms (*N* = 220)

		EC Use		
Outcomes	*n* (%)	Ever (*n* = 120)	Never (*n* = 100)	*P*-value^†^
Cough				
Frequent cough	31 (14.1)	27 (22.5)	4 (4.0)	< 0.001^‡^
Chronic cough > 3 months	28 (12.7)	16 (13.3)	12 (12.0)	0.768
Phlegm				
Frequent phlegm	34 (15.5)	24 (20.0)	10 (10.0)	0.041
Chronic phlegm > 3 months	37 (16.8)	22 (18.3)	15 (15.0)	0.510
Episodes of cough and phlegm > 3 weeks	27 (12.3)	19 (15.8)	8 (8.0)	0.078
Wheezing				
Ever sound wheezy or whistling	47 (21.4)	35 (29.2)	12 (12.0)	0.002
Episode of wheezing attack	34 (15.5)	22 (18.3)	12 (12.0)	0.196
Shortness of breath (mMRC grades)				
Grade 0 (no breathlessness)	140 (63.6)	66 (30.0)	74 (33.64)	0.005
Grade 1 (mild)	25 (11.4)	14 (6.4)	11 (5.0)	
Grade 2 (moderate)	24 (10.9)	14 (6.4)	10 (4.5)	
Grade 3 (severe)	21 (9.5)	17 (7.7)	4 (1.8)	
Grade 4 (very severe)	10 (4.6)	9 (4.1)	1 (0.5)	
Chest cold and illnesses				
Chest cold	73 (33.2)	47 (39.2)	26 (26.0)	0.039
Chest illnesses during the past 3 years	96 (43.6)	17 (14.2)	12 (12.0)	0.636
Eyes				
Ever sore or irritated	83 (37.7)	51 (42.5)	32 (32.0)	0.110
Eye irritation during the past month	57 (25.9)	34 (28.3)	23 (23.0)	0.369
Nose				
Ever sore or irritated	69 (31.4)	43 (35.8)	26 (26.0)	0.118
Nose irritation during the past month	43 (19.6)	25 (20.8)	18 (18.0)	0.598

^†^ The *P*-value was calculated by Chi-square test

^‡^ The *P*-value was calculated by Fisher’s exact test

**Fig. 2:**
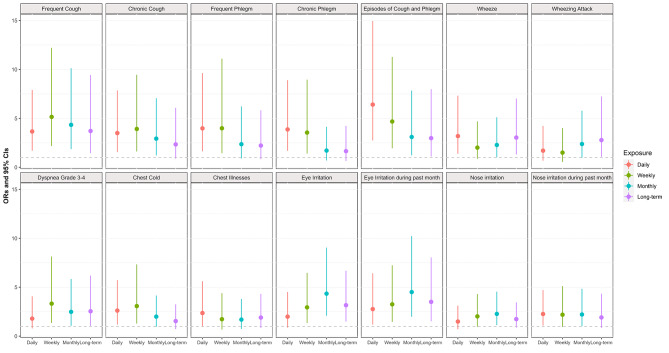
Adjusted odds ratios (ORs) and 95% CIs in respiratory symptoms associated with daily, weekly, monthly, and long-term use of EC. Models were adjusted for age, sex, race, education, and smoking status. Circle symbols indicate the ORs

Figure 2 shows the associations of respiratory symptoms with the short-term and relatively long-term use of EC. The subjects with use of EC had prevalent respiratory symptoms than never users: the past 7-day EC use was associated with frequent cough (OR: 5.15, 95% CI: 2.18 to 12.21), chronic cough (OR: 3.92, 95% CI: 1.62 to 9.45), frequent phlegm (OR: 3.99, 95% CI: 1.44 to 11.10), chronic phlegm (OR: 3.55, 95% CI: 1.41 to 8.96), episodes of cough and phlegm > 3 weeks (OR: 4.68, 95% CI: 1.94 to 11.28), mMRC grade 3–4 dyspnea (OR: 3.32, 95% CI: 1.35 to 8.13), chest cold (OR: 3.07, 95% CI: 1.29 to 7.33), eye irritation (OR: 2.94, 95% CI: 1.34 to 6.47), and nose irritation (OR: 2.02, 95% CI: 0.95 to 4.30), respectively. We also found relatively long-term effects of the past 90 days EC use on wheeze (OR: 3.04, 95% CI: 1.31 to 7.03), wheeze attack (OR: 2.78, 95% CI: 1.07 to 7.24), mMRC grade 3–4 dyspnea (OR: 2.54, 9% CI: 1.05 to 6.18), eye irritation (OR: 3.16, 95% CI: 1.49 to 6.68), and eye irritation during the past month (OR: 3.50, 95%CI: 1.52 to 8.04), respectively. The past 30-day EC use remained significant with higher odds in association with these symptoms.

### Sensitivity analyses

We conducted a sensitivity analysis to examine if the results are similar among subjects without respiratory disease (*n* = 169, 77% of 220). We found that the significant associations between short-term use of EC and respiratory symptoms remained robust among individuals without respiratory diseases (Supplementary Table 2). We also found the independent effect of cigarette smoking as expected (Supplementary Table 3). When we assessed the effect of EC use among never smokers (*n* = 96), the effect sizes were similar or greater but wide confidence intervals due to the small sample size (Supplementary Table 4). We also analyzed the data using ‘EC regular users’, defined as EC use in the last 1 day, 7 days, 30 days, and 90 days, and found the effects of EC use on respiratory symptoms (Supplementary Table 5). We analyzed the data after excluding the participants who ever tested positive for COVID-19 (*n* = 20) at the time of baseline and found similar associations (Supplementary Table 6).

## Discussion

In this repeated measures study using the application of mobile app, we found that short-term use of EC (the past seven days) was associated with a range of respiratory symptoms including frequent and chronic cough, phlegm production, shortness of breath, chest cold, and irritation of eye and nose in adults in the U.S. In addition, we found that relatively long-term use of EC over the past 90 days was associated with an increased prevalence of wheeze and shortness of breath. The use of mobile technology was feasible to recruit and collect data entirely remotely on a smartphone app for epidemiologic study.

Few studies have examined the short-term and long-term effects of EC use on a range of respiratory symptoms, as well as eye and nose irritation. Although direct comparison is limited, studies reported that the use of EC was associated with self-reported wheezing and other related respiratory symptoms in US adults aged 18, [26] chronic respiratory symptoms, defined having at least one of the following: cough, sputum production, or breathlessness during the past 3 months in the US adults aged 18, [12] increased risk of self-reported asthma, chronic obstructive pulmonary symptoms (COPD), and asthma-COPD overlap syndrome (ACOS) among never smokers in the US adults aged 18, [24] obstructive lung function impairment among Canadian adults aged 45–85 years, [27] and the development of wheezing-related respiratory symptoms in the US young adults aged 18–24 years [28]. However, most studies are cross-sectional design [12, 26, 27] which are susceptible to reverse causation. Xie and colleagues (2022) reported the longitudinal association between the use of EC and the increased risk of self-reported wheezing in the chest (OR: 1.51, 95% CI: 1.06–2.14). [28] However, respiratory symptoms other than self-reported wheezing in association with short-term and long-term use of EC has not been well characterized in the general population. The present study findings add to the preceding literature by demonstrating that the use of EC was longitudinally associated with the respiratory symptoms such as chronic cough, phlegm, shortness of breath (also referred to as dyspnea) and wheezing, which are hallmark signs of respiratory illness such as COPD.

The mechanisms by which EC may cause a range of respiratory symptoms likely depend on the chemical constituents of EC products and the level of inhaled toxic chemicals. EC fluid (e-liquid) contains multiple toxic chemicals such as nicotine, carbonyls, volatile organic compounds (VOCs), particles, trace metal elements, flavorants, and microbial toxins [6–10, 29] that have been implicated with adverse respiratory health outcomes [29–32]. It is also possible that inhaling user-manipulated EC products by adding other toxic substances [e.g., tetrahydrocannabinol (THC) or cannabidiol (CBD)] may cause respiratory symptoms. In the general US adult population, individuals who had ever vaped a manipulated EC product by adding marijuana concentrates, marijuana waxes, THC, or hash oils had an increased prevalence of self-reported respiratory symptoms (such as wheezing and dry cough) over the past 12 months compared to those who had ever vaped EC product alone [33].

We acknowledge several limitations. First, all data are self-reported, like many prior observational studies, and may be prone to misclassification. Our study is a prospective mobile app survey and uses real-time recording of multiple respiratory symptoms from the participants themselves [34]. Mobile app-based self-reporting is a valid tool in the assessment of symptoms in patients with bipolar disorder, [35] cost-efficient, can be simply implemented, and capable to collect amount of data automatically, reducing the risk for errors with manual processes. Second, we recruited volunteer participants via social media which may not be generalizable to all population, although using social media as a recruitment may be effective and efficient strategy. Third, the modest sample size may make estimates unstable. To maximize statistical power, we used repeated measurements and adopted generalized linear mixed models to detect the associations. Fourth, despite the inclusion of important covariates in our models, the potential for uncontrolled confounding cannot be ruled out. Although our repeated measures longitudinal study could find short-term and long-term effects of EC use on respiratory outcomes, the present study did not have information on user behavior factors such as puff topography (e.g., puff duration, puff number) and e-liquid characteristics (e.g., flavorants). Further study is needed to incorporate the behavioral patterns and health outcomes. Lastly, although we found similar associations when we excluded the participants with diagnosed with COVID-19 at the time of baseline, it is possible that some subjects may have COVID-19 symptoms but may not be diagnosed with COVID-19. In that case, the individual with COVID symptom may less likely use EC products and has respiratory symptoms, which may cause underestimation of our observed effect size. Further longitudinal studies using mobile app surveys are needed to confirm our findings, and the contribution of mobile technology development to assess potential chronic effects of vaping while monitoring diseases is expected.

## Conclusion

In this study, we found the association between short-term and relatively long-term use of EC and increased prevalence of a range of respiratory symptoms in the adult population. Our study platform illustrates the value of prospective collection of data on the evaluation of respiratory health outcomes in the setting of potential toxic inhalation exposures.

### Electronic supplementary material

Below is the link to the electronic supplementary material.

Supplementary Material 1

## Data Availability

The data used in this study is not publicly available, but they are available from the corresponding author upon reasonable request.

## Abbreviations

App: application
ATS-DLD: American Thoracic Society division of lung disease
COPD: chronic obstructive pulmonary symptoms
EC: e-cigarette
EVALI: e-cigarette, or vaping, product use-associated lung injury
GLMM: generalized linear mixed model
mMRC: modified Medical Research Council
OR: odds ratio
VHS: Vaping and Health Study
