# Supplementary material for: Vaping habits and respiratory symptoms using a smartphone app platform
Source: BMC Public Health. 2024 Jul 30;24:2047. doi: 10.1186/s12889-024-19439-0 (PMC11289986; doi:10.1186/s12889-024-19439-0)

**Supplementary Table 1.** Terminology and question

| **Terminology** | **Question** |
| --- | --- |
| Frequent cough | Do you usually cough as much as 4 to 6 times a day, 4 or more days out of the week? |
| Chronic cough | Do you usually cough like this [at all during the rest of the day or at night] on most days for 3 consecutive months or more during the year? |
| Frequent phlegm | Do you usually bring up phlegm like this as much as 4 to 6 times a day, 4 or more days out of the week? |
| Chronic phlegm | Do you bring up phlegm like this [at all during the rest of the day or at night] on most days for 3 consecutive months or more during the year? |
| Episode of cough and phlegm | Have you had periods or episodes of increased cough and phlegm lasting for 3 weeks or more each year? |
| Ever sound wheezy or whistling | Does your chest ever sound wheezy or whistling? |
| Episode of wheezing attack | Have you ever had an attack of wheezing that has made you feel short of breath? |
| Shortness of breath Grade Ⅰ | Are you troubled by shortness of breath when hurrying on the level or walking up a slight hill? |
| Shortness of breath Grade Ⅱ | Do you have to walk slower than people of your age on the level because of breathlessness or have to stop for breath when walking at your own pace on the level? |
| Shortness of breath Grade Ⅲ | Do you ever have to stop for breath after walking about 100 yards (or after a few minutes) on the level? |
| Shortness of breath Grade Ⅳ | Are you too breathless to leave the house or breathless on dressing or undressing? |
| Chest cold | If you get a cold, does it usually go to your chest? |
| Chest illnesses | During the past 3 years, have you had any chest illnesses that have kept you off work, indoors at home, or in bed? |
| Eyes ever sore or irritated | Are your eyes ever sore or irritated? |
| Eye irritation during the past month | Have you had this problem during the past month? |
| Nose ever sore or irritated | Is your nose ever sore or irritated? |
| Nose irritation during the past month | Have you had this problem during the past month? |

**Supplementary Table 2**. Adjusted odds ratios (ORs) and 95% CIs in respiratory symptoms associated with daily use of EC among overall subjects (*n*=220) and subjects without respiratory diseases (*n*=169). Models were adjusted for age, sex, race, education, and smoking status. Models were adjusted for age, sex, race, education, and smoking status.

| **Outcomes** | **All subjects (*n* =220)** | **Subjects without respiratory disease (*n* =169)** |
| --- | --- | --- |
|  | **OR (95% CI)** | **OR (95% CI)** |
| **Cough** |  |  |
| Frequent cough | 3.66 (1.70, 7.90) | 3.85 (1.42, 10.45) |
| Chronic cough > 3 months | 3.50 (1.56, 7.85) | 3.34 (1.22, 9.17) |
| **Phlegm** |  |  |
| Frequent phlegm | 3.98 (1.64, 9.62) | 2.23 (0.68, 7.30) |
| Chronic phlegm > 3 months | 3.87 (1.69, 8.90) | 3.45 (1.17, 10.18) |
| **Episodes of cough and phlegm > 3 weeks** | 6.41 (2.75, 14.95) | 5.22 (1.78, 15.30) |
| **Wheezing** |  |  |
| Ever sound wheezy or whistling | 3.19 (1.39, 7.32) | 4.80 (1.65, 13.91) |
| Episode of wheezing attack | 1.70 (0.68, 4.23) | 3.42 (0.99, 11.80) |
| **Shortness of breath** |  |  |
| mMRC grade 3,4 | 1.61 (0.71, 3.65) | 1.82 (0.65, 5.12) |
| **Chest cold and illnesses** |  |  |
| Chest cold | 2.61 (1.19, 5.73) | 2.23 (0.89, 5.61) |
| Chest illnesses during the past 3 years | 2.36 (0.99, 5.61) | 3.53 (1.13, 11.06) |
| **Eyes** |  |  |
| Ever sore or irritated | 1.99 (0.88, 4.51) | 2.73 (1.02, 7.33) |
| Eye irritation during the past month | 2.76 (1.18 (6.42) | 4.22 (1.43, 12.52) |
| **Nose** |  |  |
| Ever sore or irritated | 1.49 (0.71, 3.11) | 1.33 (0.55, 3.22) |
| Nose irritation during the past month | 2.26 (1.08, 4.70) | 1.44 (0.56, 3.73) |

**Supplementary Table 3.** Adjusted odds ratios (ORs) and 95% CIs in respiratory symptoms associated with smoking. Models were adjusted for age, sex, race, education, and EC use.

| **Symptoms** | **Smoking status** | Past 1-day | Past 7-days | Past 30-days | Past 90-days |
| --- | --- | --- | --- | --- | --- |
|  |  | OR (95% CI) | OR (95% CI) | OR (95% CI) | OR (95% CI) |
| **Cough** |  |  |  |  |  |
| Frequent cough | Current | 4.47 (1.64, 12.15) | 3.66 (1.25, 10.69) | 3.05 (1.08, 8.61) | 2.11 (0.69, 6.45) |
|  | Past | 2.75 (0.94, 8.04) | 1.83 (0.59, 5.66) | 1.44 (0.48, 4.30) | 1.15 (0.36, 3.69) |
|  | Never | reference | reference | reference | reference |
| Chronic cough | Current | 1.25 (0.50, 3.16) | 1.52 (0.55, 4.21) | 0.97 (0.36, 2.65) | 0.70 (0.23, 2.06) |
|  | Past | 0.94 (0.34, 2.59) | 0.89 (0.31, 2.58) | 0.66 (0.23, 1.85) | 0.59 (0.20, 1.76) |
|  | Never | reference | reference | reference | reference |
| **Phlegm** |  |  |  |  |  |
| Frequent phlegm | Current | 4.23 (1.47, 12.20) | 6.74 (1.94, 23.44) | 4.79 (1.48. 15.48) | 3.73 (1.18, 11.78) |
|  | Past | 1.24 (0.36, 4.27) | 1.72 (0.44, 6.66) | 1.21 (0.33, 4.42) | 1.05 (0.30, 3.73) |
|  | Never | reference | reference | reference | reference |
| Chronic phlegm | Current | 2.18 (0.76, 6.26) | 1.88 (0.66, 5.33) | 1.43 (0.52, 3.92) | 1.33 (0.45, 3.89) |
|  | Past | 1.60 (0.52, 4.94) | 0.92 (0.31, 2.74) | 0.88 (0.31, 2.45) | 0.95 (0.32, 2.77) |
|  | Never | reference | reference | reference | reference |
| **Episodes of cough and phlegm > 3 weeks** | Current | 2.18 (0.76, 6.26) | 2.01 (0.70, 5.76) | 1.95 (0.61, 6.24) | 1.67 (0.48, 5.76) |
|  | Past | 1.60 (0.52, 4.94) | 1.43 (0.49, 4.16) | 2.25 (0.74, 6.90) | 2.21 (0.69, 7.05) |
|  | Never | reference | reference | reference | reference |
| **Wheezing** |  |  |  |  |  |
| Ever sound wheezy or whistling | Current | 3.75 (1.36, 10.34) | 4.43 (1.62, 12.15) | 3.71 (1.36, 10.14) | 2.23 (0.81, 6.19) |
|  | Past | 3.41 (1.21, 9.63) | 2.66 (0.96, 7.34) | 2.53 (0.93, 6.90) | 1.83 (0.67, 4.97) |
|  | Never | reference | reference | reference | reference |
| Episode of wheezing attack | Current | 1.05 (0.36, 3.03) | 1.56 (0.50, 4.86) | 1.15 (0.39, 3.34) | 0.84 (0.27, 2.63) |
|  | Past | 1.96 (0.71, 5.40) | 1.47 (0.49, 4.44) | 1.42 (0.53, 3.86) | 1.16 (0.41, 3.30) |
|  | Never | reference | reference | reference | reference |
| **Shortness of breath** |  |  |  |  |  |
| Grade 3-4 | Current | 3.43 (1.26, 9.30) | 3.77 (1.23, 11.56) | 2.24 (0.77, 6.49) | 2.29 (0.76, 6.91) |
|  | Past | 2.68 (0.93, 7.70) | 2.75 (0.88, 8.56) | 2.03 (0.71, 5.77) | 1.96 (0.66, 3.57) |
|  | Never | reference | reference | reference | reference |
| **Chest cold and illnesses** |  |  |  |  |  |
| Chest cold | Current | 2.11 (0.87, 5.13) | 2.66 (0.97, 7.26) | 2.28 (0.94, 5.51) | 2.04 (0.85, 4.89) |
|  | Past | 1.68 (0.67, 4.20) | 1.79 (0.66, 4.83) | 1.90 (0.81, 4.45) | 1.80 (0.79, 4.09) |
|  | Never | reference | reference | reference | reference |
| Chest illnesses during the past 3 years | Current | 0.90 (0.34, 2.40) | 1.20 (0.42, 3.44) | 1.15 (0.44, 3.01) | 1.11 (0.42, 2.93) |
|  | Past | 1.55 (0.60, 4.04) | 1.69 (0.62, 4.63) | 1.63 (0.66, 4.02) | 1.47 (0.60, 3.57) |
|  | Never | reference | reference | reference | reference |

**Supplementary Table 4.** Adjusted odds ratios (ORs) and 95% CIs in respiratory symptoms associated with EC use among never smokers. Models were adjusted for age, sex, race, and education.

| Symptoms | Past 1-day | Past 7-days | Past 30-days | Past 90-days |
| --- | --- | --- | --- | --- |
|  | OR (95% CI) | OR (95% CI) | OR (95% CI) | OR (95% CI) |
| **Cough** |  |  |  |  |
| Frequent cough | 13.3 (1.08, 162.3) | 11.46 (1.25, 104.93) | 11.2 (1.38, 90.94) | 14.59 (1.70, 125.08) |
| Chronic cough | 1.45 (0.10, 19.97) | 3.76 (0.41, 34.51) | 1.92 (0.30, 12.36) | 2.17 (0.30, 15.54) |
| **Phlegm** |  |  |  |  |
| Frequent phlegm | 3.55 (0.18, 70.04) | 4.04 (0.16, 102.67) | 2.23 (0.18, 28.26) | 2.28 (0.18, 28.12) |
| Chronic phlegm | 1.15 (0.08, 16.61) | 1.43 (0.10, 20.48) | 0.76 (0.08, 7.62) | 1.02 (0.10, 10.92) |
| **Episodes of cough and phlegm > 3 weeks** | 3.54 (0.26, 48.48) | 10.45 (1.54, 70.95) | 6.14 (0.88, 43.05) | 8.24 (0.98, 69.59) |
| **Wheezing** |  |  |  |  |
| Ever sound wheezy or whistling | 2.72 (0.14, 51.34) | 2.42 (0.21, 27.39) | 9.11 (0.95, 87.47) | 10.24 (1.18, 88.70) |
| Episode of wheezing attack | 4.68 (0.36, 60.68) | 0.94 (0.06, 15.53) | 2.21 (0.26, 18.57) | 4.09 (0.47, 35.83) |
| **Shortness of breath** |  |  |  |  |
| Grade 3-4 vs. Grade 0-2 | 3.54 (0.25, 51.14) | 21.24 (2.22, 203.44) | 3.66 (0.62, 21.70) | 5.42 (0.87, 33.91) |
| **Chest cold and illnesses** |  |  |  |  |
| Chest cold | 24.15 (1.82, 320.99) | 7.58 (0.99, 57.88) | 3.97 (0.73, 21.43) | 2.14 (0.33, 13.65) |
| Chest illnesses during the past 3 years | 13.41 (1.24, 144.91) | 5.19 (2.22, 203.44) | 2.95 (0.46, 18.78) | 1.85 (0.25, 13.64) |
| **Eyes** |  |  |  |  |
| Ever sore or irritated | 1.69 (0.11, 26.09) | 11.95 (1.5, 95.47) | 7.93 (1.34, 47.00) | 6.90 (1.06, 45.07) |
| Eye irritation during the past month | 1.78 (0.15, 21.09) | 7.48 (1.26, 44.35) | 8.89 (1.98, 40.02) | 9.05 (1.77, 46.29) |
| **Nose** |  |  |  |  |
| Ever sore or irritated | 2.04 (0.19, 21.35) | 2.65 (0.46, 15.17) | 2.93 (0.63, 13.61) | 4.30 (0.92, 20.14) |
| Nose irritation during the past month | 2.90 (0.35, 23.78) | 3.59 (0.69, 18.77) | 2.05 (0.42, 9.95) | 1.95 (0.33, 11.42) |

**Supplementary Table 5**. Adjusted odds ratios (ORs) and 95% CIs in respiratory symptoms associated with EC regular use. Models were adjusted for age, sex, race, education, and smoking.

| **Symptoms** | **EC regular use** |  | **Past 90-days EC use** | |
| --- | --- | --- | --- | --- |
|  | **OR (95% CI)** | ***P*-value** | **OR (95% CI)** | ***P*-value** |
| **Cough** |  |  |  |  |
| Frequent cough | 4.06 (1.74, 9.49) | 0.0016 | 3.71 (1.46, 9.43) | 0.0067 |
| Chronic cough | 2.00 (0.91, 4.41) | 0.0865 | 2.34 (0.90, 6.08) | 0.0845 |
| **Phlegm** |  |  |  |  |
| Frequent phlegm | 2.26 (0.91, 5.59) | 0.0807 | 2.22 (0.84, 5.83) | 0.1085 |
| Chronic phlegm | 1.96 (0.79, 4.91) | 0.1513 | 1.65 (0.65, 4.23) | 0.2956 |
| **Episodes of cough and phlegm > 3 weeks** | 2.98 (1.22, 7.30) | 0.0184 | 2.98 (1.11, 7.98) | 0.032 |
| **Wheezing** |  |  |  |  |
| Ever sound wheezy or whistling | 2.39 (1.07, 5.37) | 0.0184 | 3.04 (1.31, 7.03) | 0.0106 |
| Episode of wheezing attack | 1.62 (0.65, 4.03) | 0.0363 | 2.78 (1.07, 7.24) | 0.0385 |
| **Shortness of breath** |  |  |  |  |
| Grade 3-4 | 1.51 (0.65, 3.53) | 0.3424 | 2.54 (1.05, 6.18) | 0.0416 |
| **Chest cold and illnesses** |  |  |  |  |
| Chest cold | 1.60 (0,74, 3.42) | 0.2322 | 1.54 (0.72, 3.25) | 0.265 |
| Chest illnesses during the past 3 years | 2.20 (0.98, 4.96) | 0.0588 | 1.90 (0.84, 4.31) | 0.125 |
| **Eyes** |  |  |  |  |
| Ever sore or irritated | 2.05 (0.99, 4.26) | 0.0568 | 3.16 (1.49, 6.68) | 0.0032 |
| Eye irritation during the past month | 2.29 (1.03, 4.05) | 0.0444 | 3.50 (1.52, 8.04) | 0.0037 |
| **Nose** |  |  |  |  |
| Ever sore or irritated | 2.04 (1.03, 4.05) | 0.0443 | 1.74 (0.88, 3.45) | 0.1141 |
| Nose irritation during the past month | 1.97 (0.89, 4.37) | 0.0967 | 1.91 (0.85, 4.33) | 0.1212 |

**Supplementary Table 6.** Adjusted odds ratios (ORs) and 95% CIs in respiratory symptoms associated with daily, weekly, monthly, and long-term use of EC among participants with excluding ever tested positive for COVID-19 at baseline. Models were adjusted for age, sex, race, education, and smoking status.

|  | **Past 1-day EC use** | **Past 7-days EC use** | **Past 30-days EC use** | **Past 90-days EC use** |
| --- | --- | --- | --- | --- |
| **Outcomes** | **OR (95% CI)** | **OR (95% CI)** | **OR (95% CI)** | **OR (95% CI)** |
| Cough |  |  |  |  |
| Frequent cough | 3.21 (1.33 to 7.72) | 4.83 (2.01 to 11.61) | 4.09 (1.73 to 9.67) | 3.60 (1.38 to 9.38) |
| Chronic cough > 3 months | 3.48 (1.33 to 9.10) | 3.67 (1.52 to 8.88) | 2.75 (1.15 to 6.60) | 2.24 (0.86 to 5.85) |
| Phlegm |  |  |  |  |
| Frequent phlegm | 3.67 (1.42 to 9.47) | 3.78 (1.39 to 10.31) | 2.16 (0.84 to 5.56) | 2.10 (0.81 to 5.44) |
| Chronic phlegm > 3 months | 3.48 (1.41 to 8.60) | 3.46 (1.39 to 8.62) | 1.71 (0.70 to 4.18) | 1.74 (0.68 to 4.40) |
| Episodes of cough and phlegm > 3 weeks | 5.69 (2.26 to 14.37) | 4.40 (1.80 to 10.75) | 3.20 (1.28 to 8.05) | 3.07 (1.14 to 8.22) |
| Wheezing |  |  |  |  |
| Ever sound wheezy or whistling | 2.70 (1.08 to 6.73) | 1.95 (0.82 to 4.60) | 2.13 (0.96 to 4.74) | 2.86 (1.24 to 6.63) |
| Episode of wheezing attack | 1.24 (0.44 to 3.51) | 1.51 (0.56 to 4.09) | 2.23 (0.91 to 5.42) | 2.67 (1.01 to 7.02) |
| Shortness of breath (dyspnea) severity |  |  |  |  |
| Grade I (Mild) | 0.88 (0.39 to 1.97) | 2.12 (0.99 to 4.54) | 1.74 (0.85 to 3.55) | 1.81 (0.87 to 3.76) |
| Grade II (Moderate) | 0.76 (0.31 to 1.88) | 1.39 (0.63 to 3.06) | 1.31 (0.63 to 2.73) | 1.19 (0.57 to 2.49) |
| Grade III (Severe) | 1.14 (0.45 to 2.86) | 2.94 (1.18 to 7.32) | 2.44 (1.02 to 5.82) | 2.38 (0.97 to 5.82) |
| Grade IV (Very severe) | 1.16 (0.35 to 3.88) | 2.38 (0.76 to 7.44) | 2.20 (0.64 to 7.57) | 2.41 (0.62 to 9.41) |
| Chest cold and illnesses |  |  |  |  |
| Chest cold | 2.27 (0.98 to 5.24) | 2.70 (1.13 to 6.47) | 1.81 (0.87 to 3.80) | 1.41 (0.66 to 3.02) |
| Chest illnesses during the past 3 years | 2.02 (0.79 to 5.14) | 1.76 (0.68 to 4.56) | 1.66 (0.74 to 3.73) | 1.80 (0.80 to 4.09) |
| Eyes |  |  |  |  |
| Ever sore or irritated | 1.63 (0.68 to 3.89) | 2.81 (1.27 to 6.20) | 4.33 (2.07 to 9.05) | 3.14 (1.48 to 6.66) |
| Eye irritation during the past month | 2.76 (1.18 to 6.42) | 2.34 (0.91 to 5.99) | 4.49 (1.95 to 10.35) | 3.43 (1.47 to 8.00) |
| Nose |  |  |  |  |
| Ever sore or irritated | 1.30 (0.59 to 2.86) | 1.87 (0.88 to 3.96) | 2.16 (1.07 to 4.33) | 1.65 (0.83 to 3.30) |
| Nose irritation during the past month | 2.09 (0.92 to 4.75) | 2.01 (0.87 to 4.66) | 2.08 (0.95 to 4.59) | 1.81 (0.79 to 4.13) |

**e-Figure 1. VHS App Development and Participant Onboarding**


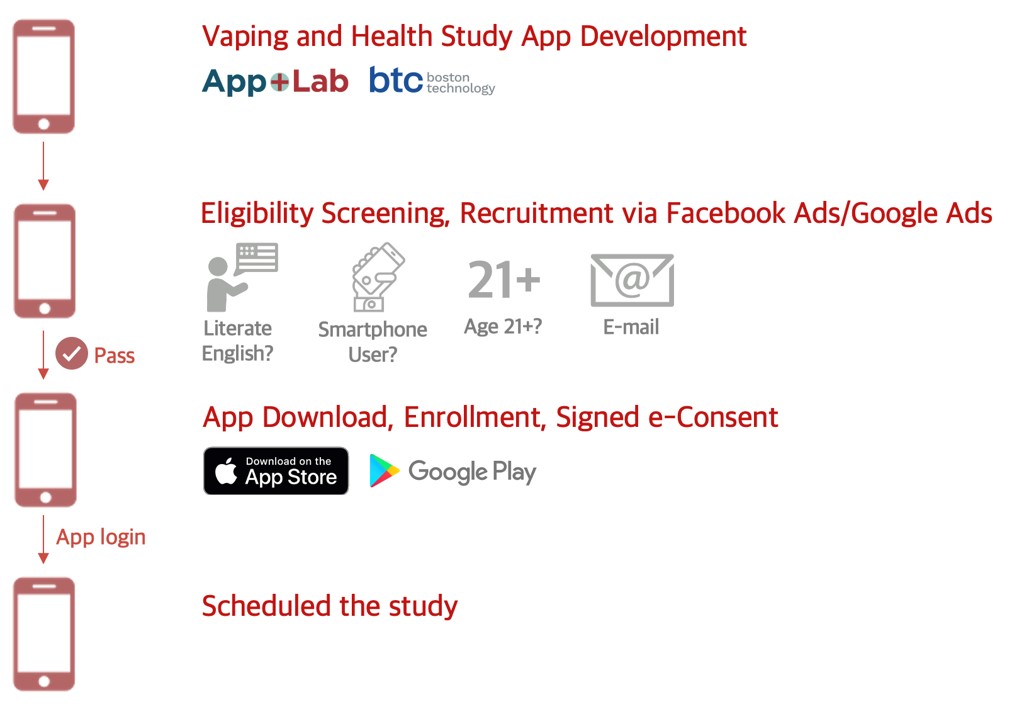


**e-Figure 2. IRB-Approved Vaping and Health Study (VHS). (A) Facebook Ad and (B) Google Ad**

1. VHS Facebook Ad

**
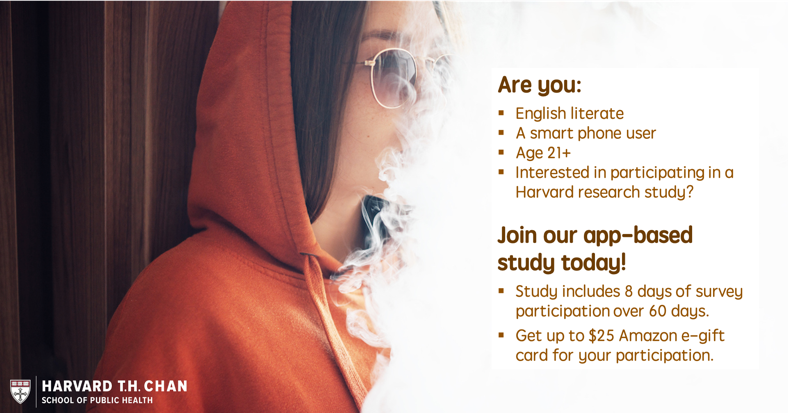
**

1. VHS Google Ad


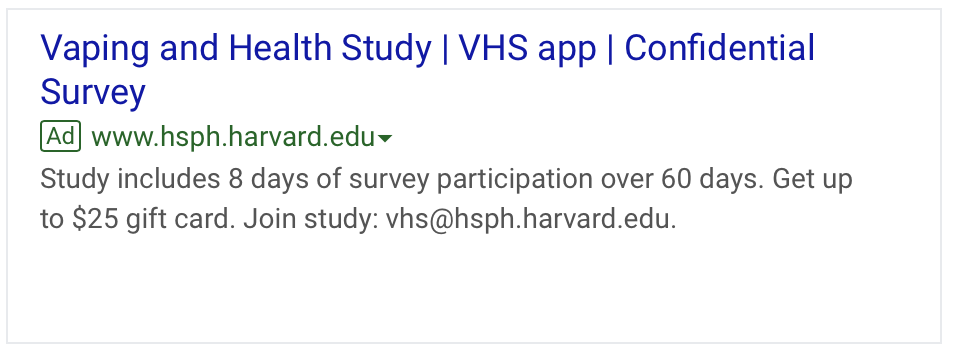

Supplement: Supplementary file 1 — Supplementary Material 1 [file 12889_2024_19439_MOESM1_ESM.docx]
